# A roadmap toward promoting and improving brain health in Europe and closing the awareness and funding gap

**DOI:** 10.1111/ene.16589

**Published:** 2025-01-15

**Authors:** Paul A. J. M. Boon, Thomas Berger, Matilde Leonardi, Tony Marson, Ulf Kallweit, Elena Moro, Antonio Toscano, Irena Rektorova, Alice Accorroni, Charlotte Scheerens, Antonia Boesch, Michael Crean, Anja Sander, Simon Lee, Claudio L. A. Bassetti

**Affiliations:** ^1^ Department of Neurology & 4Brain Ghent University Hospital Ghent Belgium; ^2^ Eindhoven University of Technology Eindhoven The Netherlands; ^3^ European Academy of Neurology Vienna Austria; ^4^ Department of Neurology and Comprehensive Center of Clinical Neurosciences & Mental Health Medical University of Vienna Vienna Austria; ^5^ Neurology, Public Health, Disability Unit and Coma Research Centre Fondazione IRCCS Istituto Neurologico C. Besta Milan Italy; ^6^ Department of Pharmacology and Therapeutics University of Liverpool Liverpool UK; ^7^ Center for Narcolepsy and Hypersomnias, Professorship for Narcolepsy and Hypersomnolence Research, Faculty of Health University Witten/Herdecke Witten Germany; ^8^ Grenoble Alpes University, CHU of Grenoble Division of Neurology, Grenoble institute of Neurosciences Grenoble France; ^9^ ERN‐NMD Center for Neuromuscular Disorders of Messina, Department of Clinical and Experimental Medicine University of Messina Messina Italy; ^10^ Brain and Mind Research Central European Institute of Technology, Masaryk University Brno Czechia; ^11^ First Department of Neurology St. Anne's University Hospital and Faculty of Medicine, Masaryk University Brno Czechia; ^12^ Hôpitaux Universitaires de Genève Geneva Switzerland; ^13^ Department of Public Health and Primary Care Ghent University, Belgium and United Nations University‐CRIS Bruges Belgium; ^14^ Department of Neurology, Inselspital University of Bern Bern Switzerland

**Keywords:** brain, brain health, burden, neurology, strategy

## Abstract

**Background and Purpose:**

The global burden of neurological diseases exceeds 43.1%, imposing a significant burden on patients, caregivers and society. This paper presents a roadmap to reduce this burden and improve brain health (BH) in Europe.

**Methods:**

The roadmap is based on the European Academy of Neurology's (EAN) five‐pillar BH strategy: advancing a global BH approach (P1), supporting policymaking (P2), fostering research (P3), promoting education (P4), and raising awareness of prevention and treatment (P5). It reviews current efforts, collaborations and future directions aligned with the WHO Intersectoral Global Action Plan (iGAP) for Neurological Disorders and suggests future initiatives and call for action.

**Results:**

1. P1: Support WHO‐iGAP through defined action points, international collaborations, in particular, the WHO BH Unit, and the EAN Brain Health Mission.
2. P2: Collaborate with 48 national neurological societies to promote National Brain Plans (NBPs), addressing local needs, and improving access to care.
3. P3: Advocate for more research funding; identify determinants of BH; develop preventive measures.
4. P4: Provide educational opportunities for neurologists, public education programs, and advocacy training, including tools to educate the public.
5. P5: Spearhead global awareness campaigns, organize public educational activities, and train BH advocates to contribute toward sustainable and long‐term public health campaigns and policy engagement.

**Conclusions:**

The paper highlights the importance of a unified approach, integrating international collaborations and local initiatives, to improve BH outcomes based on the WHO‐iGAP, and support sustainable development goals, in particular SDG 3: Good Health and Well‐being and SDG 4: Quality Education.

## INTRODUCTION

Neurological diseases are highly prevalent, affecting millions of European citizens. Conditions such as migraine and other chronic headaches, Alzheimer's disease and other cognitive disorders, epilepsy, stroke, multiple sclerosis, and Parkinson's disease are common. Altogether, disorders affecting the nervous system have recently been estimated to cause a global burden of 43% and affect more than 1/3 of the global population [1]. On top of that, mental disorders in 2019 affected 13% of the global population, though it must be noted that this only refers to 12 mental disorders. Taken together disorders of the brain and nervous system have a massive impact [2].

Brain disorders impose a significant burden on individuals, caregivers, households, healthcare systems, and economies worldwide. They can cause varying degrees of disability and impairment, affecting cognitive, emotional and affective function, mobility, communication, and quality of life. The profound impact on quality of life includes physical limitations, cognitive decline, emotional distress, stigma, social isolation, and discrimination. Disorders of the nervous system are the largest cause of disability in Europe [3]. Many of these diseases are chronic and progressive, leading to long‐term disability and the need for ongoing care and support. Caregivers and families often experience high levels of burden, stress, and burnout due to the physical, mental and financial demands of providing care, emotional support, and assistance with daily activities [4]. Neurological disorders affect men and women differently. Sex and gender are associated with differences in risk and prevalence of several of the most prevalent neurological diseases, diagnostic management and treatment outcomes [5]. Moreover, women fulfil the role of caregiver for neurological patients more often than men, which may negatively impact their health and economic situation.

**FIGURE 1:**
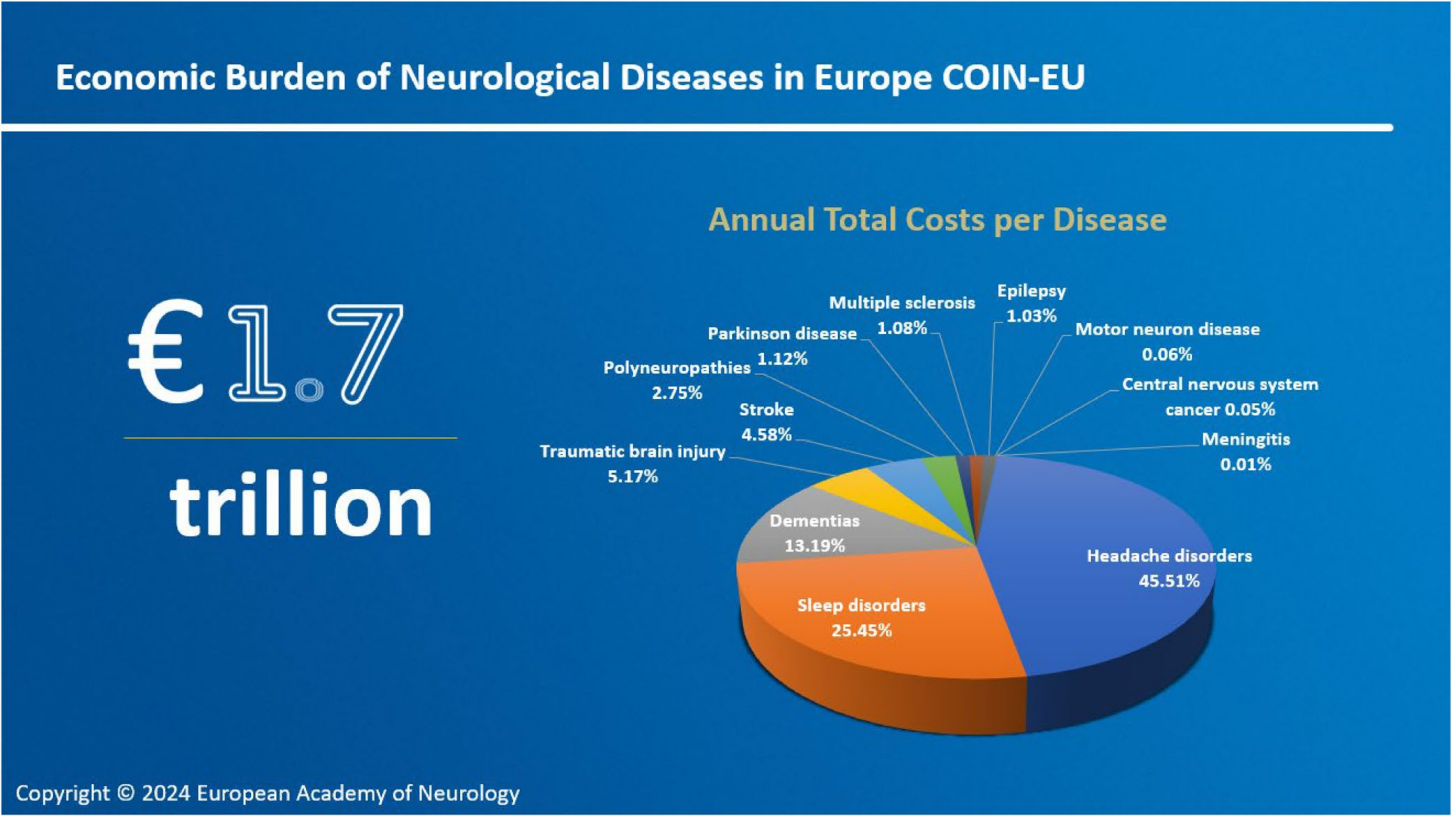
Economic burden of neurological diseases in Europe COIN‐EU [5].

Additionally, neurological diseases impose a significant economic burden on individuals, households, families, employers and society. The costs associated with medical care, rehabilitation, lost productivity, and caregiving for individuals with neurological diseases are high and increasing. In Europe, the total costs of neurological disorders in 2020 were estimated to amount to €1,7 trillion, which is more than the combined costs of cardiovascular disease, cancer, and diabetes [6] (Figure 1).

Our aim is to optimize the brain health of all throughout their lifetimes and by doing so, to reduce the prevalence and burden caused by these diseases. In addition to reducing healthcare costs, long‐term economic gains and huge boosts in well‐being can be achieved by investing in brain health [7].

## NO *HEALTH* WITHOUT BRAIN HEALTH

According to the WHO, “Brain health” is the state of brain functioning across cognitive, sensory, social‐emotional, behavioural and motor domains, allowing a person to realise their full potential over the life course, irrespective of the presence or absence of disorders [8]. Maintaining brain health typically involves adopting a healthy lifestyle, engaging in activities that stimulate the brain, and taking measures to prevent the occurrence of neurological diseases and their devastating symptoms such as cognitive decline.

The importance of neurological disorders as a global health imperative has been accepted by the World Health Assembly with the adoption of the Intersectoral Global Action Plan for Epilepsy and other Neurological Disorders (iGAP) in 2022 [9]. The iGAP is an ambitious 10‐year plan important on a European and global scale because it addresses key factors that will lead to increased awareness and targeted actions relevant to neurological disorders and brain health in member states. The WHO‐iGAP aims to address the burden of epilepsy and other neurological disorders and improve access to care and treatment for affected individuals, families, and communities worldwide, especially in low‐resource settings where access to care may be limited. IGAP also aims to promote awareness and advocacy for epilepsy and other neurological disorders, reducing stigma, increasing understanding, and mobilizing support for better care and services. It encourages collaboration and coordination among different sectors, including health, education, and social services, to holistically address the complex needs of individuals with epilepsy and other neurological disorders. Health systems need to be strengthened by training healthcare providers to improve diagnostic and treatment services and integrating mental and neurological health services into primary care as well as promoting the importance of general neurology [10]. Risk factors and age‐specific preventive measures need to be defined and promoted at all levels. The promotion of solutions for leading a brain‐healthy lifestyle in all age groups will play an important role.

The interdependency of brain health and the United Nations Sustainable Development Goals (UNSDG) is also important to highlight and support. While all UNSDG are impacting brain health in some ways, EAN would focus its efforts mainly on those where it can be meaningful and have a significant impact such as SDG 3: Good Health and Well‐being and SDG 4: Quality Education.

## NO *WEALTH* WITHOUT BRAIN HEALTH

In a post‐industrial era, the brains of citizens are the main resource of countries, especially where natural resources are limited. This “brain capital” is the most important driver of individuals and entire economies and a precondition for human progress [11]. Equity and equality, in particular gender equality in society is positively related to brain health. While education and lifelong learning are vital for fully exploiting and maintaining brain capital, so is maintaining optimal brain health through the entire life span. Healthy brain aging will lead to maintained and prolonged productivity for the economy and relief for health systems [12].

## STRATEGY

The European Academy of Neurology (EAN) defined the basis of a brain health strategy with the position paper ‘One brain, one life, one approach’ [13], laying out the main tasks to improve brain health during the entire lifespan along five strategic pillars, in line with the overall strategy of EAN:

1. Contribute to a global and international brain health approach: Collaborative actions with international organizations on a global and European level.
2. Support international and national policymaking: Collaborative actions in individual countries working with national neurological societies and other relevant national stakeholders.
3. Foster neurological and brain health research: Focus on identifying novel determinants of brain health.
4. Organize and provide education of relevant stakeholders: Focus on brain health literacy in the general public.
5. Raise public awareness of prevention and treatment: European‐level initiatives.

This contribution to the ongoing debate on the urgent need to improve awareness, enhance research efforts, and increase funding for neurological disorders has been paralleled by publications from other representative organizations in Europe and beyond, such as the European Brain Council (SEBRA, Shared European Brain Research Agenda) [14], the American Academy of Neurology [15], the World Federation of Neurology [16] and initiatives by other stakeholders such as patient organizations, industries, and private organizations. The need for a holistic approach to brain health, addressing both neurological and mental disorders, is emphasized, considering them “two sides of a coin.”

## A ROADMAP TO BRAIN HEALTH IN EUROPE AND BEYOND

Despite the increased interest and publication volume related to the definition of brain health and different avenues to improve brain health, a more concrete roadmap is needed to reduce the burden of neurological disorders and promote brain health in Europe in the coming years. This paper aims to define such a roadmap for use at the European and national levels by the neurological community and all relevant stakeholders committed to brain health. This roadmap for brain health is based on the five strategic pillars previously defined in the EAN Brain Health Strategy published in 2020 and gives an overview of what EAN aims to achieve in the upcoming years in order to contribute, in a sustainable manner, to global efforts to improve brain health for all throughout the life course.

In doing so, all actions outlined in this paper are also intended to support the strategic objectives and targets of the WHO‐iGAP. The indicators employed by WHO to monitor and assess the progress of iGAP implementation are closely observed by EAN as it assesses the progress of its brain health activities. In order to do so, EAN is reviewing its priorities on a regular basis and ensuring that EAN priorities and goals of the Brain Health Strategy are aligned with WHO‐iGAP and UNSDG, which are closely related to brain health and EAN actions.

### Pillar 1: Contribute to a global and International Brain Health Approach

The EAN will support the WHO‐iGAP on epilepsy and other neurological diseases by pursuing the five strategic objectives (strengthen policy prioritization and governance; provide effective, timely and responsive diagnosis, treatment and care; implement strategies for promotion and prevention; foster research and information systems; strengthen public health approach to epilepsy) and concrete action points derived from that strategy as defined by WHO. EAN will also support the UNSDG by approaching brain health holistically, including planning, protecting and preserving brain health at all societal levels. EAN will become a WHO‐Europe non‐state actor to ensure awareness of the importance of neurological/brain health, which is currently mainly focused on mental health.

In addition to contributions by international organisations like the World Federation of Neurology (WFN) and International League Against Epilepsy (ILAE) & International Bureau for Epilepsy (IBE), EAN will provide additional tools for the WHO‐iGAP toolkit, which was launched in July 2024 [17].

EAN has recently launched the Brain Health Mission (BHM), which includes partners from different brain‐related and other medical specialties [18]. The BHM aims to become a very inclusive platform for promoting and improving brain health beyond the traditional medical and patient‐representative ecosystem in Europe. At the time of publication, the BHM counts over 25 member organizations. The BHM will be transparently governed by the executive body (BHM Steering Committee), including appointed and elected representatives from all members. Regular meetings and biannual Brain Health Summits will ensure steady action and proper follow‐up of brain health‐promoting activities on a European level.

One major priority is unifying the different initiatives working on brain health. The current fragmentation can delay the progress of implementing WHO‐iGAP, and concrete actions promoting brain health beyond the brain health community are still scarce. EAN will actively work with European Union agencies and directorates, such as DG Health, to promote neurological (brain) disorders as a distinct disease group beyond the NCD category. The current inclusion of neurological disorders in NCD is not in line with the massive disease burden and hampers awareness and recognition, specifically by policymakers and funding agencies. EAN actions to improve the “status” of neurological disorders in the EU will be directly sponsored and led by EAN in collaboration with umbrella organizations such as the European Brain Council (EBC).

### Pillar 2: Support International and National policymaking

EAN has a strong collaboration with its 48 National Neurological Societies (NNS) and more than 10 international corresponding member societies, including over 45,000 neurologists. EAN is organizing six‐monthly NNS Forum meetings to systematically inform national neurological member societies on ongoing activities, share ideas and best practices, and follow‐up on the development and implementation of brain health policies.

To date, only a few European countries have a National Brain Plan (NBP) [19, 20]. EAN will work with all NNS to prepare, create, and promote NBPs which should address the local needs of health professionals and people with brain disorders and be easily understandable and accessible to the lay public. NBPs should also provide specific directions and guidelines for improved diagnosis and treatment of brain disorders, for appropriate provision of neurological services and easier access to neurological and mental care, and for addressing general and preventive challenges. This includes regulations to control substances negatively impacting brain health, promoting brain‐healthy environments in schools, workplaces, and communities, and supporting early childhood development programs.

### Pillar 3: Foster neurological and brain health research

During the last decade, brain research has seen a significant increase in volume and quality, resulting in improved insights into the mechanisms of many frequent and rare brain disorders. Patients with highly prevalent and chronic neurological disorders such as migraine, Alzheimer's disease, epilepsy, and multiple sclerosis can now be treated with more efficacious and safer treatments. However, the total expenditure of governments, academia, and funding agencies on neurological research is not proportional to the massive burden of neurological disorders. According to SEBRA, the European Union has spent €500 M annually on brain research between 2007 and 2019 [21]. It is estimated that less than 10% of the total biomedical research budget of €8 B is spent on neurological research, compared to 40% burden of neurological disorders [22, 23]. In the United States, the National Institutes of Health (NIH) spend (only) 20% of their total budget of $ 48B on brain research [24]. Assuming that only half of brain research resources are spent on neurological research—the other half being spent on mental health research—this represents a dramatic research funding gap that urgently needs to be decreased.

In recent years there have been significant steps to increase funding within Europe and to outline the priorities. SEBRA, published in 2022, was closely followed by the EAN Strategic Research Agenda for Neurology [25]. These papers aimed to set the main priorities for brain research and, neurology in particular, in the coming years. The forthcoming European Partnership for Brain Health is also a welcome development in this regard, with many of the organisations involved in the production of SEBRA likely to participate [26]. However, it is crucial that the consortium be maximally inclusive, and allow for substantial and meaningful input from EAN and other crucial clinical stakeholders.

While research on understanding disease mechanisms and genetic factors will be pivotal for the future development of treatments and strategies to maximize the brain health of future generations, it is also vital to undertake research that will identify other biological, environmental or social determinants of brain health and develop strategies and policies that promote brain health. An evidence base that allows to develop and implement a Brain Health Check urgently needs to be developed. This is another key priority for EAN. In the course of 2024, an EAN‐supported Strategic Brain Health Chair will be launched at Ghent University, Belgium. It is likely that other academical Brain Health Chairs will be established in other European countries. Furthermore, research on identifying the size of the disease burden in Europe such as EAN Economic Burden of Neurological Diseases projects in general neurology and the most prevalent neurological disorders are another key priority for EAN. The results of these studies are expected to become available in 2025.

### Pillar 4: Develop education strategies and provide education to relevant stakeholders at all relevant levels

#### Education of neurologists and psychiatrists

The European Academy of Neurology (EAN) focuses heavily on the education of neurologists through various platforms, including congresses, clinical and science schools, teaching courses, fellowships, and online learning opportunities. EAN collaborates with the European Union of Medical Specialists Section of Neurology (UEMS‐SN) to regularly update and systematically implement the European postgraduate training requirements for neurologists [27]. Additionally, EAN is developing a pre‐graduate neurology curriculum aimed at harmonizing the education of medical students across Europe to be published by 2025. Active collaboration with the European Psychiatric Association (EPA) and other European societies for medical specialists actively working with neurological patients will be sought, preferably within the context of the Brain Health Mission.

#### Addressing Sub‐specialties and General Neurology Education

To prevent the fragmentation of neurology into sub‐specialties and address the shortage of neurologists in many countries, EAN emphasizes the importance of educating general neurologists. It maintains close partnerships with various neurological sub‐specialties to ensure the highest level of education across the field. EAN also collaborates with other medical specialties such as the European Society of Cardiology and the European Psychiatric Association to promote interdisciplinary education at their events [10].

#### Brain Health Advocacy and Public Education

EAN has initiated a brain health advocacy training program to empower neurologists to advocate for neurology and brain health [28]. This program includes various educational activities like sessions at the annual EAN congress, podcasts, and online learning content. EAN works with patient organizations to assist with patient advocacy training programs. This program is part of the WHO‐iGAP toolkit.

#### Educational Programs for the Public

EAN plans to organize educational activities focusing on brain health for the general public, leveraging events such as the annual EAN congress as a kick‐off for ongoing national initiatives. These initiatives aim to extend beyond one‐time events, creating lasting impacts within communities.

#### Brain Health Educational Programs in Schools

EAN provides blueprints for low‐budget brain health educational programs in primary and secondary schools across Europe. Programs like the “Brain Health School Challenges” have been implemented in Austria and Finland, aiming to integrate brain health education into the school curriculum to raise awareness and reduce stigma around neurological and mental health issue [29].

#### Incorporating Brain Health into National and Community Initiatives

EAN supports the creation of national brain health plans that incorporate preventive measures and promote brain‐healthy lifestyles. These plans include public health campaigns that encourage healthy behaviours such as regular exercise, a balanced diet, and effective stress management.

#### Collaboration and Support for Non‐specialists

Recognizing the growing need for caregivers due to the ageing population, EAN also focuses on providing accessible education to non‐neurologists, including therapists, nurses and general practitioners. This effort includes collaborative training and education to enhance care for individuals with neurological conditions. Enhancing early diagnosis, particularly within primary care, and ensuring a clinical pathway that provides patients with the right treatment at the right time, and crucially contact with the appropriate specialist.

#### Development of Postgraduate Programs

EAN collaborates with academic institutions to develop postgraduate and Master/Certificate of Advanced Studies (CAS) programs in Brain Health. The first international University CAS (Certificate of Advanced Studies) on Brain Health was launched in 2024 at the University of Bern, Switzerland, with the support of the EAN and other organizations. These programs will equip clinicians, scientists, and other health professionals with advanced skills in promoting and implementing brain health [30].

### Pillar 5: Raise Public awareness

#### Public Awareness Campaigns

EAN participates in international public awareness campaigns such as Brain Awareness Week and World Brain Day and supports disease‐specific initiatives like Purple Day for epilepsy. EAN aims to enhance the visibility of neurological health issues and promote public and policy engagement.

## THE EUROPEAN ACADEMY OF NEUROLOGY *CALLS FOR ACTION* TO STAKEHOLDERS TOWARD BETTER BRAIN HEALTH IN 2024–2026 (FIGURE 2)

**FIGURE 2:**
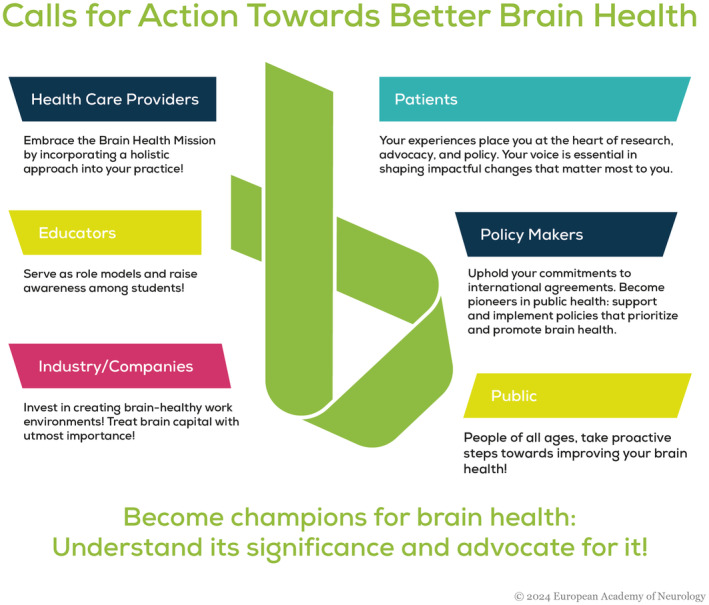
Calls for action toward better brain health.

### Neurologists and psychiatrists, child neurologists, neuro‐rehabilitators, nurses, therapists of neurological patients

*Become champions for brain health!* Understand its significance and advocate for it! Educate yourself on what constitutes brain health and leverage your knowledge to promote awareness about the importance of prevention, the necessity of ongoing research, and the holistic nature of brain health. Engage in conversations about brain health with colleagues, patients, companies, government officials, and the general public. Your efforts are crucial in shaping a future where brain health is prioritized and maintained.

### Health care providers

*Embrace the Brain Health Mission by incorporating a holistic approach into your practice!* Understand that brain health encompasses more than just the absence of disease; it involves the promotion of overall well‐being. By integrating this perspective into your daily work, you can contribute significantly to the prevention of neurological and psychiatric disorders and enhance the quality of life for your patients.

### Patients

*Patients, you are the key stakeholders in any brain health effort*. You are the main driver and justification for health care providers. Your contribution to health policy development, research, innovation, advocacy and public awareness is invaluable. Health care providers and all other stakeholders should work closely with you and your patient organisations to achieve the goals outlined in this call for action.

### Public

*People of all ages, take proactive steps toward improving your brain health!* Adopt healthy lifestyle practices as outlined in the inner layer of the “protect” figure. Empower yourself by keeping your brain healthy: quit smoking, maintain a healthy blood pressure level, be physically active, maintain a healthy weight, get enough sleep, engage in positive relationships, and manage blood sugar.

Advocate for your health by requesting referrals to neurologists or psychiatrists when necessary. Utilize available resources to stay informed and educate those around you about the various strategies for preventing neurological and psychiatric diseases. Understand the importance of consulting specialists to maintain optimal brain health.

### Educators

*Serve as role models and raise awareness among students!* Incorporate brain health (both mental and neurological) into your curriculum wherever possible! This integration can occur across various subjects such as biology, sports, and philosophy. By educating the younger generation about brain health, you are fostering a culture that values and prioritizes mental and neurological well‐being.

### Policy makers

*Become public health pioneers!* Support and implement policies that promote brain health! Collaborate with neurologists, psychiatrists, neuroscientists, healthcare providers, public health experts and patient organizations to ensure comprehensive brain health strategies. Increase public spending in neurological research, particularly clinical and patient‐centred research. In addition, invest in research focused on prevention to build a future where neurological and psychiatric diseases can be mitigated or delayed. Implement international agreements such as the WHO‐iGAP and prioritize brain health in policy‐making processes.

### Industry/Companies

*Invest in creating brain‐healthy work environments!* Treat brain capital with utmost importance! Support research initiatives aimed at understanding and enhancing brain health. By fostering a workplace culture that prioritizes mental and neurological well‐being, companies can contribute to the overall health of their employees and, in turn, enhance productivity and innovation.

### Governments/Countries

*Honour your commitments to international agreements!* lntegrate brain health into national policies as agreed in the WHO‐iGAP. Engage with neurologists, psychiatrists, healthcare providers, and patient organizations to develop and implement effective brain health strategies. Take urgent initiatives to decrease the dramatic funding gap for neurological research, making use of the recently published EAN Strategic Research Agenda for Neurology [24], that defines clinically relevant and patient‐centred research priorities. Invest in research focused on prevention to secure a future where neurological and psychiatric diseases are less prevalent, ultimately ensuring a healthier population.

### The ‘Brain Bubble’ brain health infographic

EAN has created an infographic showing the three layers of brain health. The infographic has been developed in 2021 and was first published in the EAN Brain Health Strategy [12].

In recent years it has been updated and now also includes:

- Responsible media consumption (under Preserve)
- Prevent infectious diseases (by, e.g., vaccination or avoidance of mosquito bites), in line with, for example, the WHO Defeating meningitis initiative [31] (Protect)
- Politics (Plan)

The three layers of brain health are strongly influenced by and interdependent on each other:

**FIGURE 3:**
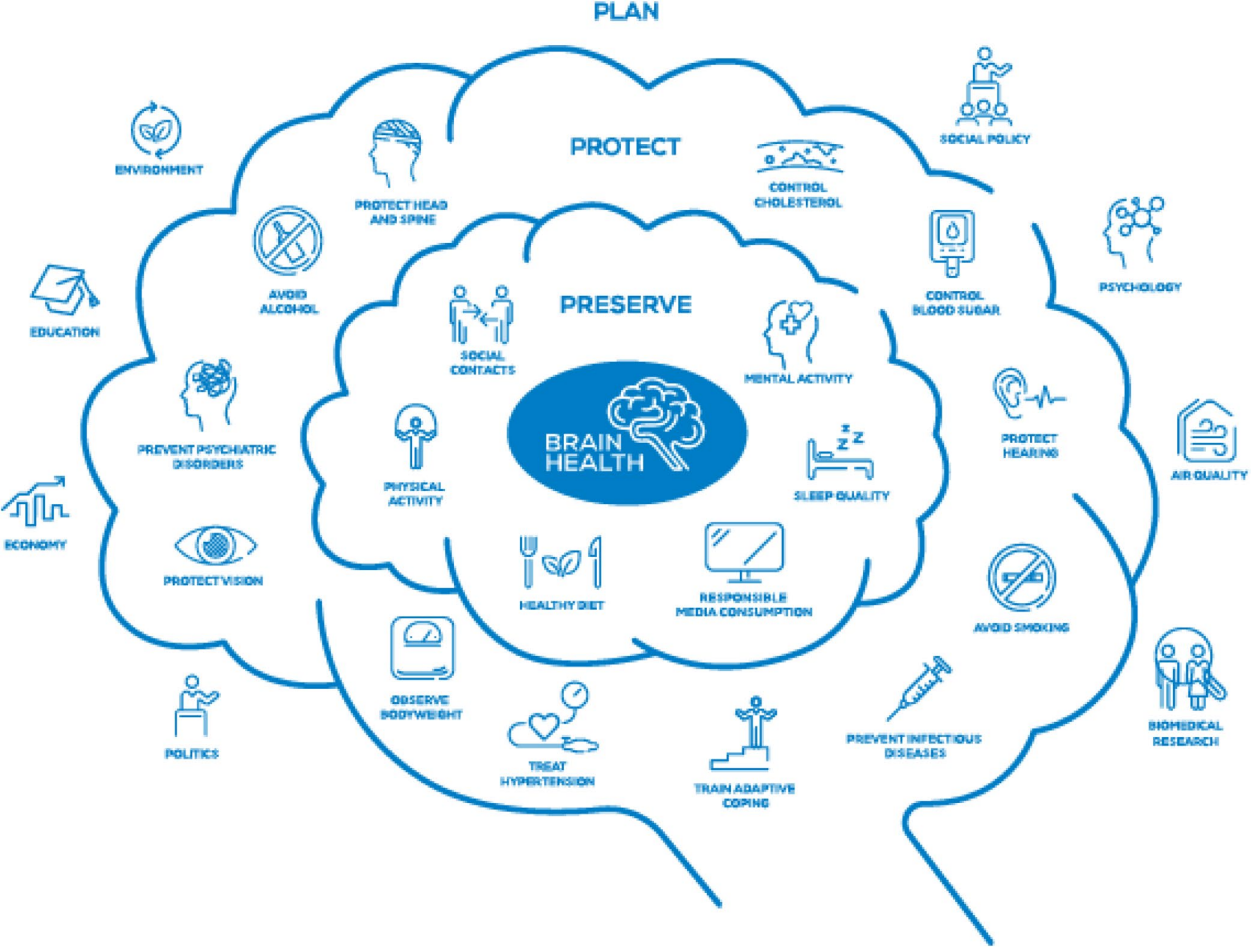
The Brain Bubble [13].

#### Layer 1: Plan

Goal: A society that is connected and approaches the health of its individuals holistically.

Items: Air quality, Biomedical research, Economy, Education, Environment, Politics, Psychology, Social Policy (Figure 3).

Listed here are external influences that seem beyond the scope for action by individuals. However, they influence everyone's life and health. By taking informed decisions these can be changed by societies and governments, and individuals' behavior can contribute to a change of culture and environment. Changing policies, governments, politicians and stakeholders can impact the future of health and well‐being of societies.

#### Layer 2: Protect

Goal: A society in which supporting HCPs can support individuals and there is investment in measures that can reduce the burden of diseases.

Items: Avoid alcohol, Avoid smoking, Control blood sugar, Control cholesterol, Observe bodyweight, Prevent infectious diseases, Prevent psychiatric disorders, Protect head and spine, Protect hearing, Protect vision, Train adaptive coping, Treat hypertension.

Protection of brain health can be achieved by individuals together with their social surroundings. Individuals are only able to take care of their brain health in an environment in which they are supported. Such support can be provided by neurologists, psychiatrists, nurses, therapists, general practitioners, governments, and teachers, but also by parents and families.

The better a society plans (see 6.9.1—Layer 1), the better individuals can be supported.

#### Layer 3: Preserve

Goal: A society in which each individual has the knowledge and ability to take care of their brain health.

Items: Healthy Diet, Mental activity, Physical activity, Responsible media consumption, Sleep quality, Social Contacts.

Listed here are measures that individuals can take on their own and that they can influence themselves. Of course, education on the impact of these measures is necessary, and a supportive environment and well‐planned, safe surroundings will be necessary for individuals to be able to take these measures.

## CONCLUSION

In this paper, we have outlined the mission and vision of EAN, the European Academy of Neurology, and its various activities and initiatives to advance the field of neurology and improve the quality of life of people with neurological disorders. We have highlighted some of the key achievements and challenges of EAN, such as its scientific congresses, educational programs, public awareness campaigns, and advocacy efforts. We have also discussed some of the future directions and opportunities for EAN, such as expanding its global collaborations, fostering interdisciplinary research, and embracing digital transformation.

We believe that EAN is a leading organisation in the field of neurology and that it has a significant impact on the scientific, clinical, and social aspects of brain health. We also recognise that EAN faces many challenges and uncertainties in the rapidly changing and complex environment of health care and research. Therefore, we encourage EAN and the neurology community to continue efforts to promote excellence, innovation, and collaboration in neurology, and to address the needs and expectations of its members, partners, and stakeholders. We hope that this paper provides a useful overview of EAN and its current contributions and plans to promote and improve Brain Health in Europe and beyond. We hope that it will inspire further dialogue and cooperation among the neurology community and all relevant stakeholders and that our calls for change and action will be followed by concrete and much‐needed actions.

## AUTHOR CONTRIBUTIONS

**Paul A. J. M. Boon:** Conceptualization; writing – original draft; methodology; supervision. **Thomas Berger:** Writing – review and editing; supervision. **Matilde Leonardi:** Writing – review and editing; supervision. **Tony Marson:** Writing – review and editing. **Ulf Kallweit:** Writing – review and editing; supervision. **Elena Moro:** Writing – review and editing; supervision. **Antonio Toscano:** Writing – review and editing; supervision. **Irena Rektorova:** Writing – review and editing; supervision. **Alice Accorroni:** Writing – review and editing; supervision. **Charlotte Scheerens:** Writing – review and editing. **Antonia Boesch:** Visualization; writing – review and editing. **Michael Crean:** Conceptualization; investigation; funding acquisition; methodology; validation; visualization; writing – review and editing; project administration; formal analysis; software; supervision; data curation; resources. **Anja Sander:** Conceptualization; writing – original draft; methodology; supervision; project administration. **Simon Lee:** Writing – review and editing; visualization. **Claudio L. A. Bassetti:** Writing – review and editing; supervision.

## Supporting information


Video S1.

## Data Availability

Data sharing is not applicable to this article as no new data were created or analyzed in this study.
