# Supplementary figures and images for: A roadmap toward promoting and improving brain health in Europe and closing the awareness and funding gap
Source: Eur J Neurol. 2025 Jan 15;32(1):e16589. doi: 10.1111/ene.16589 (PMC11735729; doi:10.1111/ene.16589)

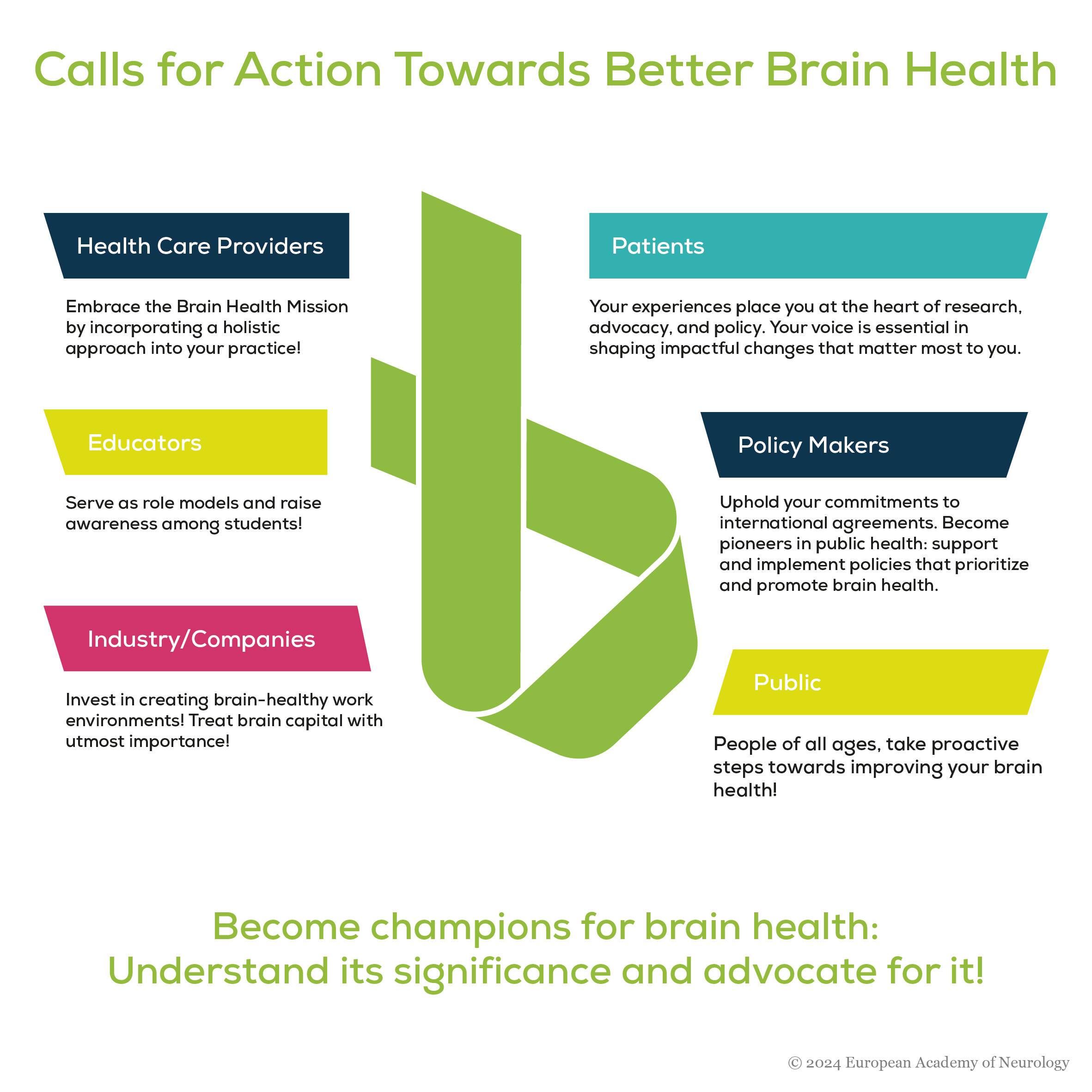

Supplement: Supplementary file 1 — Video S1. [file ENE-32-e16589-s001.png]
